# Propionate Protects Haloperidol-Induced Neurite Lesions Mediated by Neuropeptide Y

**DOI:** 10.3389/fnins.2018.00743

**Published:** 2018-10-15

**Authors:** Minmin Hu, Peng Zheng, Yuanyi Xie, Zehra Boz, Yinghua Yu, Renxian Tang, Alison Jones, Kuiyang Zheng, Xu-Feng Huang

**Affiliations:** ^1^Jiangsu Key Laboratory of Immunity and Metabolism, Xuzhou Medical University, Jiangsu, China; ^2^Illawarra Health and Medical Research Institute, School of Medicine, University of Wollongong, Wollongong, NSW, Australia

**Keywords:** antipsychotic drug, haloperidol, neurite impairment, propionate, neuropeptide Y

## Abstract

Haloperidol is a commonly used antipsychotic drug for treating schizophrenia. Clinical imaging studies have found that haloperidol can cause volume loss of human brain tissue, which is supported by animal studies showing that haloperidol reduces the number of synaptic spines. The mechanism remains unknown. Gut microbiota metabolites, short chain fatty acids including propionate, are reported to have neuroprotective effect and influence gene expression. This study aims to investigate the effect and mechanism of propionate in the protection of neurite lesion induced by haloperidol. This study showed that 10 μM haloperidol (clinical relevant dose) impaired neurite length in human blastoma SH-SY5Y cells, which were confirmed by using primary mouse striatal spiny neurons. We found that haloperidol impaired neurite length were accompanied by a decreased neuropeptide Y (NPY) expression, but no effect on GSK3β signaling. Importantly, this project research found that propionate was capable of protecting against haloperidol-induced neurite lesions and preventing NPY reduction. To confirm this finding, we used specific siRNAs targeting NPY which blocked the protective effect of propionate on haloperidol-induced neurite lesions. Furthermore, since NPY is regulated by the nuclear transcription factor CREB, we measured pCREB that was decreased by haloperidol and was normalized by propionate. Therefore, propionate has a protective effect against pCREB-NPY mediated haloperidol-induced neurite lesions.

## Introduction

Antipsychotic drugs are the primary therapeutic agents used to treat schizophrenia and its allied mental disorders (Huang and Song, 2018). Among them, haloperidol is the first-generation antipsychotic drug and widely used to treat schizophrenia patients (Tardy et al., 2014). Haloperidol acts on the dopamine D2 receptors (D2R) and controls psychotic symptoms including hallucinations, delusions, and aggressiveness (Jafari et al., 2012; Dold et al., 2015). However, it can also cause various side effects including extrapyramidal syndrome, tardive dyskinesia, and cerebrovascular events (Hufner et al., 2015). In brain morphology, chronic application of haloperidol has been reported to reduce brain volume (Ho et al., 2011). A number of studies have suggested that chronic or accumulative haloperidol administration can decrease synaptic spines and induce apoptosis (Frost et al., 2010; Nandra and Agius, 2012). A chronic application of haloperidol induces neurite lesion reported in human, animal, and cell-based studies (Kelley et al., 1997; Dorph-Petersen et al., 2005; Critchlow et al., 2006; Ho et al., 2011). A meta-analysis revealed that higher daily haloperidol intake in patients resulted in greater cortical gray matter reduction (*Z*, -2.31, *p* = 0.02) (Vita et al., 2015). Animal study shows that macaque monkeys treated with haloperidol for 17 to 27 months have a reduced brain weight by 8–11%, and these reductions were consistent across a number of brain areas (Dorph-Petersen et al., 2005). Another study shows that chronic administration of haloperidol at ∼0.35 mg/kg at 2-week intervals for 1 year significantly reduces neuronal cytoskeleton and spine-associated proteins in the cortices of rhesus monkey, where are rich in dopamine innervation and are implicated in the psychopathology of schizophrenia (Lidow et al., 2001). Therefore, there is an urgent need to search for a way to protect antipsychotic drug-induced neurite lesion.

Our previous study shows that haloperidol decreases neuropeptide Y (NPY) mRNA expression in the rat brain after haloperidol treatment (Huang et al., 2006). NPY is highly co-expressed in GABAergic neurons and is found to be a modulator of the neuroplasticity, neurotransmission, and memory (Gotzsche and Woldbye, 2016). Given these evidence, we have investigated whether or not NPY was involved in haloperidol-induced neurite lesion.

Short chain fatty acid (SCFA) including acetate, propionate, and butyrate are the metabolites produced by gut microbiome fermentation on dietary fiber. SCFA can enter the circulation via monocarboxylate transporters, cross the blood–brain barrier, and thereby enter the central nervous system (Pierre and Pellerin, 2005; Kekuda et al., 2013). More and more evidence show that SCFA regulate cell metabolism (Canfora et al., 2015), neurotransmitter synthesis and release (DeCastro et al., 2005; Shah et al., 2006), epigenetics (Yamawaki et al., 2012), and immune function (Correa-Oliveira et al., 2016). In particular, propionate and butyrate act as the histone deacetylases inhibitors (HDACi). HDACi regulates brain gene expression, improving the healthy state of patients suffering from Parkinson’s disease, depression, and schizophrenia (Galland, 2014). However, the neurite protective, at high concentrations, propionate has also been reported to induce autism-like behavioral changes in rats (Macfabe, 2012). Collectively, these reports suggest that propionate may play an important role in neural function. Our study investigated whether or not propionate may be used to prevent haloperidol-induced neurite lesions. Furthermore, we have investigated the CREB-NPY signaling pathway in mediating the neurite protective effect of propionate in haloperidol-induced neurite lesion.

## Materials and Methods

### Cell Culture and Treatments

The undifferentiated human SH-SY5Y neuroblastoma cell line were grown in Dulbecco’s modified Eagle’s medium (DMEM)-F12 supplemented with 1% penicillin–streptomycin and 10% heat-inactivated fetal bovine serum (FBS) from Bovogen Biologicals (Victoria, Australia). For differentiation, cells were seeded in culture plates coated with MaxGel^TM^ ECM (E0282, Sigma Aldrich, Syndey). In the following day, media was removed and replaced with 10 μM retinoic acid (RA, R2625; Sigma-Aldrich) in DMEM-F12 with 1% FBS. Haloperidol (MP Biomedicals, Solon, OH) was dissolved in 100% dimethyl sulfoxide (Sigma-Aldrich). Sodium propionate was purchased from Sigma-Aldrich. Cells were treated with media containing either haloperidol or haloperidol with different concentrations of propionate. The neurite length was acquired in real time every 6 h for 24 h using IncucyteZoom Machine and analyzed with the Neuro Track software (Sartorius, Michigan).

### Gene Transfection

The siNPYs (siNPY_001: 5′CAGACCTCTTGATGAGAGA3′; siNPY_002: 5′CGCTGCGACACTACATCAA3′; siNPY_003: 5′GAGGACATGGCCAGATACT3′) and respective negative control (NC) were synthesized (RiboBio, Guangzhou) and dissolved in the DEPC H_2_O. Transfections of siRNAs were performed with the Lipofectamine 2000 (Invitrogen, Carlsbad, CA) following the manufacturer’s instructions. Medium was changed to the differentiation medium containing various treatments 6 h later.

### Primary Striatal Neuronal Culture

Cultured striatal neurons were harvested from postnatal days 0 to 3 of C57Bl mice. Briefly, striatal neurons were gently dissociated with a plastic pipette after digestion with 0.5% trypsin (GIBCO, Los Angeles) at 37°C for 30 min. Neurons were cultured in neurobasal medium (GIBCO) containing B27 supplement (GIBCO) and 20 mM glutamine (Sigma Aldrich). After 24 h of culture, 5-fluoro-2′-deoxyuridine (Sigma Aldrich) was added at a final concentration of 10 μM to repress the growth of glial cells. Cultures were maintained at 37°C in a humidified 5% CO_2_ incubator for 7 days (DIV7) prior to treatments. All experimental procedures for primary cell culture were approved by the Animal Ethics Committee, University of Wollongong, Australia, and complied with the Australian Code of Practice for the Care and Use of Animals for Scientific Purposes.

### Western Blot

After 24 h treatments, cells were harvested with lysis buffer containing NP40 (Sigma-Aldrich), Protease Inhibitor Cocktail (Sigma-Aldrich), 1 mM PMSF (Sigma-Aldrich), and 0.5 mM β-glycerophosphate (Sigma-Aldrich). Total protein concentrations were determined by DC-Assay (Bio-Rad, Sydney) and detected with a SpectraMax Plus384 absorbance microplate reader (Molecular Devices, Sunnyvale, CA). Samples were heat-treated in Laemmli buffer at 95°C, loaded to 10% SDS-PAGE gels (Bio-Rad) for fractionation, and then transferred into Immun-Blot TM PVDF membranes (Bio-Rad). The blocking buffer consisted of 5% slim milk in TBST. The membranes were incubated with NPY (sc-28943, Santa Cruz Biotechnology, Santa Cruz), phospho-GSK3β(Ser9) (#9323s, Cell Signaling Technology), and β-Catenin (#8480s, Cell Signaling Technology) antibodies in TBST containing 1% milk at 4°C overnight. Secondary antibodies were anti-rabbit IgG conjugated with horseradish peroxidase (Santa Cruz Biotechnology). For visualization, we used ECL detection reagents and obtained high resolution images with Amersham Gel Imager (GE Healthcare life Sciences).

### Immunofluorescence Assay

Primary striatal neurons were grown to approximately 70% confluence on glass coverslips and treated with either negative control, haloperidol, haloperidol + propionate, or propionate for 24 h before being fixed in 4% formaldehyde for 15 min. Neurons were washed in PBS, and permeabilized with 0.3% Triton X-100 in PBS for 10 min. After blocking with 5% normal donkey serum for 1 h at room temperature, primary antibodies of MAP2 (M4403-2ML, Sigma-Aldrich), NPY, or GAD67 (MAB5406, Millipore, Bedford) were applied in 1% donkey serum in PBS at 4°C overnight. This was followed by incubation in a secondary antibody Alexa Fluor 488-conjugated donkey anti-mouse IgG (Invitrogen, Carlsbad, CA) or Alexa Fluor 488-conjugated donkey anti-rabbit IgG (Invitrogen, Sydney), Alexa Fluor^TM^ 647-conjugated donkey anti-mouse IgG (Invitrogen), and Alexa Fluor 568 Phalloidin (A12380, Invitrogen) at room temperature for 2 h.

For SH-SY5Y cells, cells were seeded in the ibidi glass bottom dish (ibidi GmbH, Germany), treated with either haloperidol, haloperidol + propionate, propionate, or nil control in differentiated medium for 24 h, and then the above steps were followed by immunofluorescence assays. We applied the primary antibodies including pCREB (sc-101662, Santa Cruz Biotechnology) and MAP2 to cells at 4°C overnight, followed by incubation in a secondary antibody cocktail of Alexa Fluor 488-conjugated donkey anti-rabbit IgG (Invitrogen) and Alexa Fluor 647-conjugated donkey anti-mouse IgG (Invitrogen) at room temperature for 2 h. Cells were viewed using 40× or 63× oil immersion objective on a DMI6500B confocal microscope (Leica, Mannheim, Germany). The neurite length and protein expression were measured using the ImageJ Software.

### Spine Morphology

Primary striatal neurons were cultured for 14 days (DIV14) and were used for spine morphology study. The procedure was similar to the Immunofluorescence assay. After blocking, neurons were incubated with Alexa Fluor^TM^ 568 Phalloidin (A12380, Invitrogen) for 1 h and washed with PBS. Neurons were viewed using a 63× oil immersion objective on a DMI6500B confocal microscope (Leica, Mannheim, Germany). The number of synaptic spines was measured using ImageJ Software.

### Statistics

SPSS program (version 21; Chicago, IL, United States) was used for statistical analysis. One-way analysis of variance (ANOVA) followed by *post hoc* Tukey’s tests was performed for multiple comparisons. Data were expressed as mean ± SEM, and *p* < 0.05 value was considered statistically significant.

## Results

### Haloperidol Inhibited Neurite Outgrowth

**FIGURE 1:**
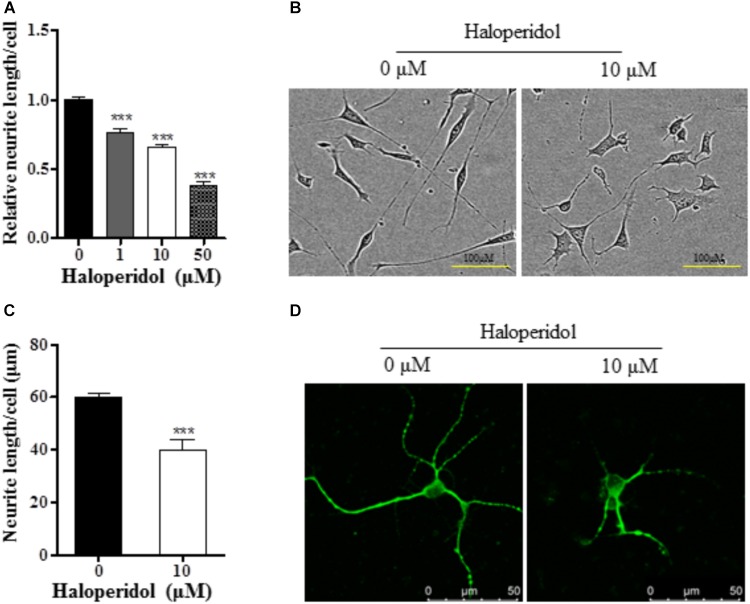
Haloperidol inhibited neurite outgrowth. **(A)** Neurite length was reduced in a dose dependent manner by 1, 10, and 50 μM haloperidol treatments in SH-SY5Y cells for 24 h (*n* = 3–4); **(B)** representative images of cells with (10 μM) and without (0 μM) haloperidol treatments in SH-SY5Y cells; **(C)** haloperidol (10 μM) decreased neurite length/cell in primary striatal neurons (*n* = 6); **(D)** primary striatal neurons stained with MAP2 antibody and imaged with immunofluorescence confocal microscope. Mean ± SEM, ^∗∗∗^*p* < 0.001.

In order to investigate the effects of haloperidol on neurite morphology, RA-induced differentiated SH-SY5Y cells were incubated with various concentrations of haloperidol (0, 1, 10, 50 μM) for 24 h. The neurite length was measured in real-time. Haloperidol treatment significantly reduced the neurite outgrowth in a dose-response manner [*F*(3,11) = 145.401, *p* < 0.001, **Figure 1A**]. The neurite length was significantly shorter compared with control group after 1, 10, and 50 μM haloperidol treatments (all *p* < 0.001). As it is known that the plasma concentration of haloperidol-treated patients is between 2 and 10 ng/ml (Volavka et al., 1995), we used 3.8 ng/ml or 10 μM concentration of haloperidol. We repeated the results with 10 μM haloperidol and confirmed its effect on neurite inhibition (**Figure 1B**). To confirm these observations, we applied the same concentration of haloperidol in primary striatal neurons. We found that haloperidol treatment at 10 μM for 24 h significantly reduced neurite length visualized by MAP2 staining (*p* < 0.001, **Figures 1C,D**).

### Haloperidol-Induced Neurite Lesions Did Not Alter GSK3β Signaling but Decreased NPY Expression

**FIGURE 2:**
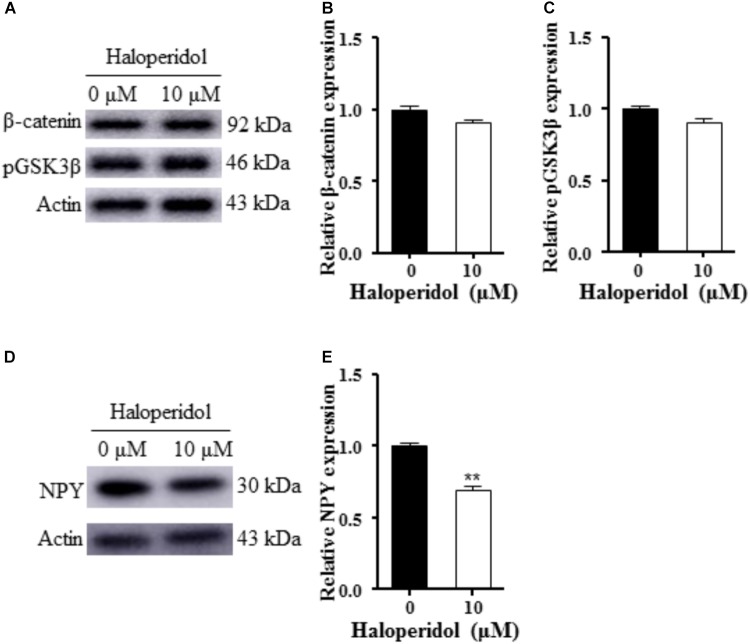
Haloperidol reduced neuropeptide Y (NPY); however, haloperidol did not change GSK3β signaling. **(A–C)** Haloperidol (10 μM) reduced the neurite length, but no changes were found in pGSK3β_Ser9_ and β-catenin signaling in SH-SY5Y cells measured by Western blot (*n* = 3); **(D,E)** haloperidol (10 μM) reduced NPY expression in SH-SY5Y cells (*n* = 3). Mean ± SEM, ^∗∗^*p* < 0.01.

We examined GSK3β signaling since this signaling pathway is involved in neurogenesis and synaptic plasticity (Cole, 2013). No changes were detected in pGSK3β and β-catenin expression after 10 μM haloperidol treatment (*p* > 0.05, **Figures 2A–C**). However, we found a significant reduction of NPY expression after 10 μM haloperidol treatment in SH-SY5Y cells (*p* < 0.01, **Figures 2D,E**). The results suggested that NPY was associated with neurite reduction induced by haloperidol.

### Propionate Prevented Neurite Lesion-Induced by Haloperidol

**FIGURE 3:**
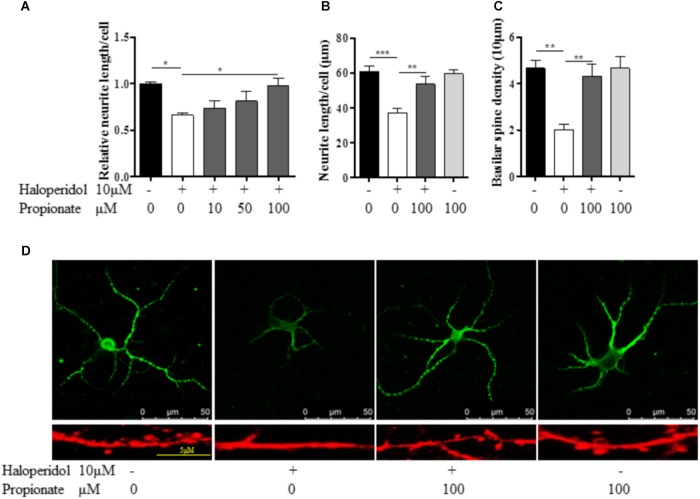
Propionate protected neurite lesions. **(A)** Neurite lesions induced by 10 μM haloperidol were protected by 100 μM propionate in SH-SY5Y cells (*n* = 3–4); **(B)** Propionate prevented the haloperidol-induced neurite lesion in primary striatal neurons (*n* = 6); **(C)** Propionate prevented the haloperidol-induced synaptic spine reduction in primary striatal neurons (*n* = 6); **(D)** The striatal neurons were stained with MAP2 antibody (Top row). Synaptic spines were stained with Alexa Fluor^TM^ 568 Phalloidin (Bottom row) and imaged with immunofluorescence confocal microscope. Cells were treated for 24 h. Mean ± SEM, ^∗^*p* < 0.05, ^∗∗^*p* < 0.01, ^∗∗∗^*p* < 0.001.

Previous studies suggest that propionate may play important roles in neurodevelopment. We tested whether or not propionate could prevent haloperidol-induced neurite deficit. We treated SH-SY5Y cells with sodium propionate at concentrations of 0, 10, 50, and 100 μM (Fasting plasma concentration in humans is about 24 μM.) for 30 min prior to 10 μM haloperidol treatment. We observed that propionate prevented neurite impairment induced by haloperidol [*F*(4,11) = 5.357, *p* = 0.012, **Figure 3A**]. Specifically, 100 μM propionate completely prevented neurite lesions induced by haloperidol (*p* < 0.05). Furthermore, we investigated if propionate could prevent neurite lesion in primary mouse striatal neurons. Our results showed that propionate prevented neurite lesion induced by haloperidol [*F*(3,20) = 11.656, *p* < 0.001, **Figures 3B,D**]. *Post hoc* analysis showed that the neurite lesions was prevented by 100 μM propionate (**Figure 3B**, *p* < 0.01). It has been reported that haloperidol can decrease the number of synaptic spines in the rat striatum (Kelley et al., 1997). We quantified the number of basilar synaptic spines. We found that propionate prevented the loss of synaptic spines-induced by haloperidol [*F*(3,20) = 9.994, *p* < 0.001, **Figures 3C,D**].

### Propionate Prevented NPY Reduction Induced by Haloperidol

**FIGURE 4:**
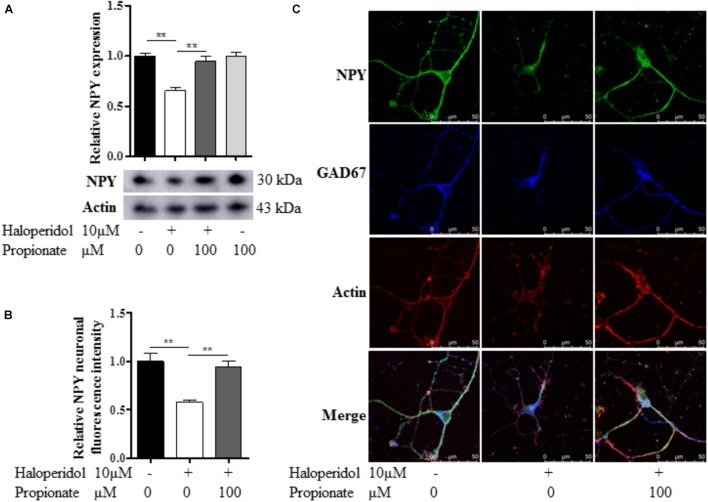
Propionate prevented the NPY reduction induced by haloperidol. **(A)** Propionate (100 μM) protected haloperidol induced NPY reduction in SH-SY5Y cells (*n* = 3); **(B)** propionate prevented NPY reduction induced by haloperidol in primary striatal neurons (*n* = 6); **(C)** these striatal NPY neurons were GAD67 positive indicating that NPY and GABA are co-localized in these neurons. The morphological appearance showed these are medium sized spiny neurons with body size of ∼10 μM in diameter. Mean ± SEM, ^∗∗^*p* < 0.01.

Since we observed that the neurons with neurite deficits induced by haloperidol have decreased NPY, we examined if propionate could prevent the reduced NPY. We found that propionate completely prevented NPY reduction induced by haloperidol in SH-SY5Y cells [*F*(3,8) = 17.502, *p* = 0.001, **Figure 4A**]. This result supported our above discovery that NPY is involved in haloperidol-induced neurite lesions and propionate prevented neurite lesions were involved in the regulation of NPY. Furthermore, we used primary mouse striatal neurons to validate our finding. Again, we found that haloperidol significantly reduced NPY, which could be prevented by propionate in the striatal neurons [F(2,15) = 12.346, *p* = 0.001, **Figures 4B,C**]. Further characterization was performed using glutamic acid decarboxylase 67 (GAD67) antibody staining for GABA synthesis (Chattopadhyaya et al., 2007). We showed these haloperidol-responding striatal neurons contained both NPY and GABA (**Figure 4C**).

**FIGURE 5:**
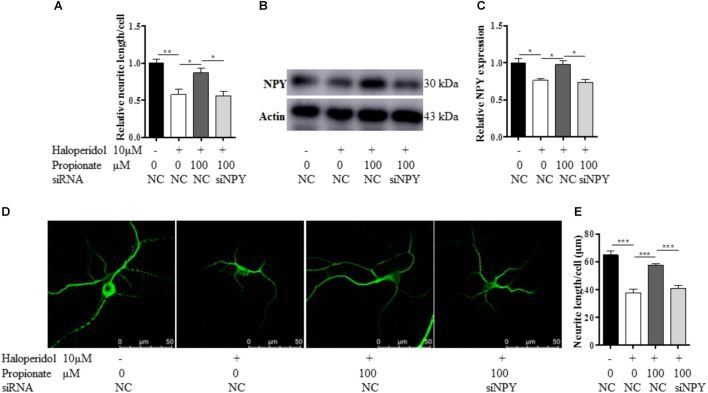
The treatment of NPY-siRNA (siNPY) eliminated the protective effects of propionate on the neurite impairment-induced by haloperidol. **(A)** The quantification of neurite length of SH-SY5Y cells treated with/without siNPY (*n* = 3); **(B,C)** the treatment of siNPY eliminated the effect of propionate in preventing haloperidol induced NPY reduction in SH-SY5Y cells (*n* = 3); **(D)** fluorescence confocal images of striatal neurons stained for MAP2, and **(E)** quantification of neurite length showed that the treatment of siNPY eliminated the effect of propionate in preventing haloperidol-induced neurite lesions in primary striatal neurons (*n* = 6). Mean ± SEM, ^∗^*p* < 0.05, ^∗∗^*p* < 0.01, ^∗∗∗^*p* < 0.001.

Furthermore, to confirm the role of NPY in propionate-induced neuroprotection against haloperidol-induced neurite lesions, we used specific siRNA to knock down NPY in SH-SY5Y cells (**Figures 5A–C**). In this case, propionate was no longer able to protect the neurite lesion-induced by haloperidol [*F*(3,8) = 11.788, *p* = 0.003, **Figure 5A**]. As shown that the neurite lesions induced by haloperidol (*p* < 0.01) was protected by 100 μM propionate (*p* < 0.05), while siNPY abolished the neural protective effects of propionate. Western blotting results showed that the NPY was reduced in NPY-siRNA treated cells compared with the cells without NPY-siRNA treatment (*p* < 0.05, **Figures 5B,C**). Similarly, propionate prevented neurite lesion in primary mouse striatal neurons but not after NPY-siRNA treatment [*F*(3,20) = 29.663, *p* < 0.001, **Figures 5D,E**]. These results indicated that propionate protective effect was mediated by NPY.

### CREB Phosphorylation Was Involved in the Neuronal Protective Effect of Propionate

**FIGURE 6:**
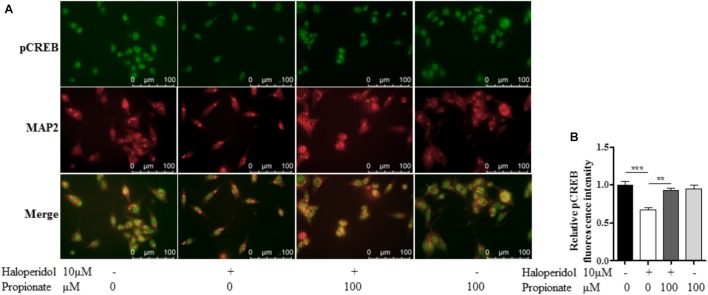
Propionate protected the reduction of CREB phosphorylation (pCREB) induced by haloperidol in SH-SY5Y cells. **(A)** Fluorescence confocal images of SH-SY5Y cells showed that haloperidol reduced pCREB which was prevented by propionate (Top row), cell contour showed by MAP2 staining (middle row), and merge (bottom row). **(B)** The quantification of pCREB immunofluorescence intensity (*n* = 6). Mean ± SEM, ^∗∗^*p* < 0.01, ^∗∗∗^*p* < 0.001.

**FIGURE 7:**
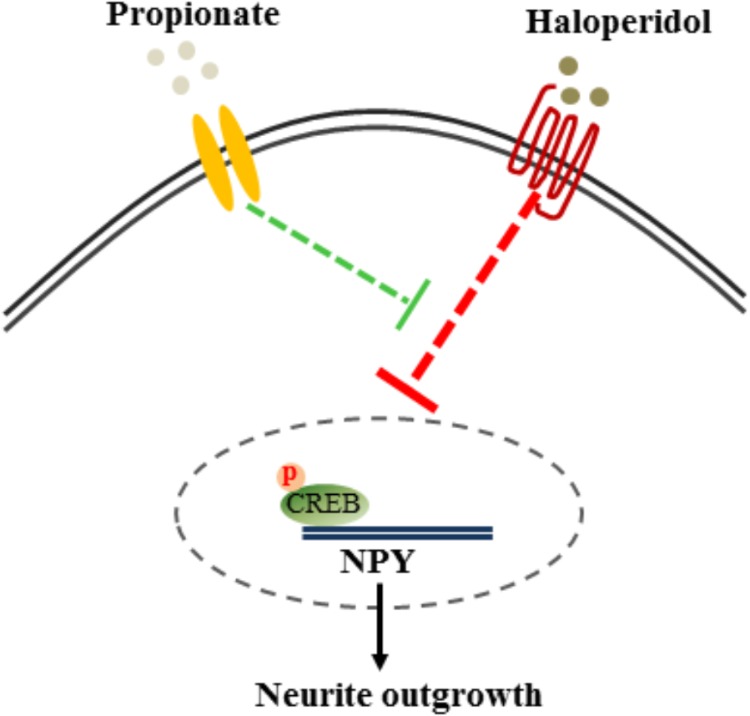
Proposed mechanism of propionate preventing haloperidol-induced neurite lesion *in vitro*. Haloperidol inhibited pCREB-NPY signaling causing neurite deficit. Propionate prevented haloperidol-induced reduction of pCREB-NPY signaling, which reversed neurite deficits.

The cAMP responsive element binding protein (CREB) is a ubiquitous transcription factor located in CRE promoter regions, which modulates the transcription of genes with cAMP responsive elements (CRE). Since it is known that CREB gene transcription factor regulates NPY expression in neurons, we investigated possible correlations between phosphorylated CREB in our propionate treated cells. As expected, we observed that haloperidol decreased CREB phosphorylation, which was prevented by propionate; however, propionate alone did not alter CREB phosphorylation [*F*(3,20) = 14.741, *p* < 0.001, **Figures 6A,B**]. These data suggested that propionate could prevent the down-regulation of CREB phosphorylation induced by haloperidol, which in turn prevented down-regulation of NPY and neurite lesions (**Figure 7**).

## Discussion

Antipsychotic drugs are widely used in treating schizophrenia and allied disorders. However, antipsychotic drug treatment could result in neurite lesion in brain areas, rich in dopamine D2R (Huang and Song, 2018). This is because D2R plays an important role in neurite growth and synaptogenesis (Jia et al., 2013) and virtually all antipsychotic drugs have D2R antagonist properties (Jafari et al., 2012; Huang and Song, 2018). Haloperidol is a typical antipsychotic drug having strong D2R antagonist property. Plasma concentrations of patients treated with haloperidol are between 2 to 10 ng/ml (Volavka et al., 1995). This study used a clinical relevant dose 3.8 ng/ml (equivalent to 10 μM; molecular weight of haloperidol is 376 g/mol), which resulted in neurite lesions in both SH-SY5Y and primary striatal neurons. A previous study has shown that 0.1 μM haloperidol treatment decreases the dendritic spine density as well as spines-enriched proteins of rat hippocampal neurons (Critchlow et al., 2006). Similarly, chronic haloperidol treatment (1.5 mg/kg daily similar to human dose) results in 58% reduction of spine density in rat striatum (Kelley et al., 1997). These data provide strong evidence that clinically used doses of haloperidol may cause neurite lesion.

Propionate is a short chain fatty acid which is a product of gut microbiome fermentation of dietary fiber (Neis et al., 2018). Short chain fatty acids can affect brain function and behavior (Selkrig et al., 2014). Our study showed that propionate prevented neurite lesions-induced by haloperidol. Previous study has suggested that propionate can have significant effects on brain neurites. For example, children with autistic spectrum disorders are characterized by elevated concentrations of propionate, which may link to exaggerated neural synaptic spine formation (Penzes et al., 2011; Frye et al., 2015). Introducing propionate into brain ventricle could induce autism-like behavioral changes in rats (MacFabe et al., 2007, 2011). Our study showed that propionate protected the neurite and synaptic spine lesion induced by haloperidol supporting the effect of propionate on promoting neurite outgrowth.

NPY is highly expressed in striatal GABAergic neurons and regulates GABA and glutamate release potentially contributing to neuroprotection, learning, and memory (Gotzsche and Woldbye, 2016). The present study showed that haloperidol decreased NPY expression in both human blastoma SH-SY5Y and primary striatal neurons. This is in consistent with our early *in vivo* study, which we have demonstrated that haloperidol decreases NPY expression in the rat amygdala and hippocampus (Huang et al., 2006). Other studies have shown that haloperidol decreases NPY in the striatum, but increases in the hypothalamus (Gruber and Mathe, 2000). The present study showed that propionate prevented haloperidol-induced NPY reduction in SH-SY5Y and striatal neurons. When we used NPY-siRNA to decrease NPY activity, we found that the protective effect of propionate against haloperidol-induced lesion in neurites was eliminated. Collectively, these results support the idea that the NPY pathway was involved in the prevention of haloperidol-induced neurite lesion by propionate.

The mechanism underlying haloperidol decreasing NPY expression is not clear. One possibility could be via CREB signaling. CREB is a transcriptional coactivator involved in the regulation of synaptic plasticity and long-term memory through activation of gene transcription (Vecsey et al., 2007). It is known that the CREB pathway regulates NPY expression (Wand, 2005). It has also been demonstrated that D2R regulates CREB. For example, D2R agonist quinpirole stimulates CREB phosphorylation by activating protein kinase C and Ca^2+^/calmodulin-dependent protein kinase (Yan et al., 1999).

In addition, GSK3β signaling pathway is involved in neurogenesis and synaptic plasticity (Cole, 2013). Previous studies have reported that haloperidol does not change the GSK3β pathway in SH-SY5Y cells and in primary hippocampal neurons (Park et al., 2009, 2011). In agreement with their studies, we did not find a change of GSK3β signaling after haloperidol treatment. Our study showed that haloperidol reduced CERB phosphorylation, which was prevented by propionate. Therefore, it is suggested that haloperidol may act on other D2R protein kinase dependent pathway to inhibit pCREB rather than the pGSK3β signaling pathway as per previous observations (Borroto-Escuela et al., 2013; Fuxe et al., 2014).

It is known that short chain fatty acids are ligands for G-protein coupled receptors GPR41 and GPR43 (Tan et al., 2014), which have no other known ligands (Tazoe et al., 2008). Until now, no GPR41 or GPR43 receptors have been reported in the brain. On the other hand, it is known that short chain fatty acids can directly enter brain and interact with neurons (Erny et al., 2015; Stilling et al., 2016). Our study showed that propionate prevented haloperidol-induced neurite lesions in a dose-dependent manner. The concentration of propionate is between 14 to 19 mM in human feces (Schwiertz et al., 2010) and 19.4 to 28.5 μM in human fasting plasma, which could vary depending on the detection method used or a person’s dietary profile (De Filippo et al., 2010). It is possible that increasing propionate by either delivering highly concentrated propionate capsule or providing selected dietary fiber may provide a possible protective effect against neurite lesion.

## Conclusion

Our study showed that haloperidol reduced neural pCREB-NPY signaling, which was involved in neurite and synaptic spine lesions. Propionate prevented haloperidol-induced neurite lesions via increased pCREB-NPY signaling *in vitro*. Further study needs to be performed to examine if propionate could protect neurite lesion induced by haloperidol *in vivo*.

## Author Contributions

XH, MH, PZ, and YX provided substantial contributions to the conception or design of the work. MH performed the acquisition and analysis of data for the work. XH, MH, ZB, YY, RT, AJ, and KZ were involved in the interpretation of data and manuscript preparation for important intellectual content. All authors have read the paper and agreed to be authors on the paper.

## Conflict of Interest Statement

The authors declare that the research was conducted in the absence of any commercial or financial relationships that could be construed as a potential conflict of interest.
